# Flavor, antimicrobial activity, and physical properties of composite film prepared with different surfactants

**DOI:** 10.1002/fsn3.1526

**Published:** 2020-06-04

**Authors:** Xin Li, Zong‐Cai Tu, Xiao‐Mei Sha, Yun‐Hua Ye, Zhong‐Ying Li

**Affiliations:** ^1^ College of Chemistry and Chemical Engineering Jiangxi Normal University Nanchang China; ^2^ National R&D Center for Freshwater Fish Processing Jiangxi Normal University Nanchang China; ^3^ Engineering Research Center for Freshwater Fish High‐value Utilization of Jiangxi Jiangxi Normal University Nanchang China; ^4^ State Key Laboratory of Food Science and Technology Nanchang University Nanchang China

**Keywords:** antimicrobial activity, composite film, different surfactants, flavor, physical properties

## Abstract

Different surfactants (lecithin, Tween‐20, and Tween‐80) were added in composite film during the preparation. Flavor, antimicrobial activity, and physical properties of ginger essential oil ‐gelatin film were investigated, in order to study the effect of surfactants on the properties of film. The flavor of GEO was not detected in the film prepared with Tween‐20 and film prepared with Tween‐80, and these two films exhibited stronger antimicrobial activity; film prepared with lecithin possessed higher value in thickness, elongation at break, water solubility, Δ*E* and opacity, lower value in water vapor property, and tensile strength; attenuated total reflectance‐Fourier transform infrared spectrum results suggested, Tween‐20 and Tween‐80 enhanced the strength of covalent bond, and lecithin weakened the strength of hydrogen bond; and the result of scanning electron microscope showed that Tween‐20 and Tween‐80 improved the dispersion of oil droplets in film. Therefore, this study suggested that surfactants had an influence on the physical properties and molecular structure of a resulting film; in addition, Tween‐20 and Tween‐80 could reduce the flavor of GEO in film, improving the antimicrobial activity of film.

## Graphical abstract

Different surfactants have different effects in the physical properties and molecular structure. The flavor of ginger essential oil was not detected in film using Tween‐20 and film using Tween‐80. The antimicrobial activity of film using Tween‐20 and film using Tween‐80 was higher than control film and film using lecithin.

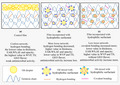

## 1 INTRODUCTION

Edible film has been gained increased focus by customers, researchers, and environmentalist, because it was safe to eat and environmentally degradable (Bourtoom, 2008; Donhowe & Fennema, 1993). Edible film could keep food water content, texture properties, color, flavor, and other properties; thus, edible film was applied as food packaging materials on meat, vegetables, and fruits to extend the shelf life of food (Dehghani, Hosseini, & Regenstein, 2017). Gelatin was an important resource of edible film (Dehghani et al., 2017), which had the characteristics of biocompatibility, film‐forming ability, and commercial availability at relatively low cost (Etxabide, Leceta, Cabezudo, Guerrero, & Caba, 2016; Zaman, Islam, Khan, & Khan, 2011). Ginger essential oil (GEO) extracted from natural plant was considered as a safe food additive, which was added into gelatin film to improve the bioactivity of gelatin film (El‐Baroty, El‐Baky, Farag, & Saleh, 2010; Silva et al., 2018; Singh et al., 2008). Adding essential oil into film would affect the properties of film, such as thickness, water vapor property (WVP), tensile strength (TS), elongation at break (EAB), color, optical, microstructural, and so on (Acosta et al., 2016; Biddeci et al., 2016; Choi, Singh, & Lee, 2016; Lee, Lee, & Song, 2015); meanwhile, the disadvantages of adding essential oil were that the undesirable flavor would affect consumer acceptance of the film (Acevedo‐Fani, Salvia‐Trujillo, Rojas‐Graü, & Martín‐Belloso, 2015; Dashipour et al., 2015; Song, Zuo, & Chen, 2018).

Surfactant had the function of provoking stable state between water phase and oil phase, forming homogeneity distribution of oil droplets (Tongnuanchan, Benjakul, & Prodpran, 2014). Essential oil should be mixed with surfactant to obtain a stable and uniform film solution due to the insolubility of oil in water (Peng, Yin, & Li, 2013; Song, Zuo, et al., 2018). Different types of surfactants were used in the preparation of composite film; Lee, Lee, Yang, and Song (2016) used Tween‐80 as surfactant during the preparation of essential oil‐protein film; Tongnuanchan, Benjakul, Prodpran, and Nilsuwan (2015) mixed palm oil and lecithin in advance, and then, the mixture was added into film solution; Prodpran, Benjakul, and Artharn (2007) selected Tween‐20 as surfactant to emulsify palm oil; Tongnuanchan et al. (2014) studied the influence of different surfactants on thermal properties of essential oil film. However, the effect of different surfactants on the physical properties of essential oil films remained not clear, especially on the flavor and antimicrobial activity. Therefore, the aim of this work was to prepare GEO film with different surfactants (hydrophilic/hydrophobic); in order to figure out the effect of surfactants on flavor, antimicrobial properties, mechanical capacities, WVP, water solubility (WS), color, light transmittance, attenuated total reflectance‐Fourier transform infrared spectrum (ATR‐FTIR), and scanning electron microscope (SEM) were applied.

## 2 MATERIALS AND METHODS

### 2.1 Materials and reagents

Lecithin (l‐α‐Phosphatidylcholine, hydrophilic‐lipophilic balance [HLB] = 4.0) was purchased from Solarbio. Dichloromethane was obtained from Aladdin. Tween‐20 (HLB = 16.7), Tween‐80 (HLB = 15.0), and glycerol were provided by Sigma‐Aldrich. GEO was obtained from Yumei Cosmetics Company. Fish gelatin with 270 Bloom (Jiliding Biotechnology Company) was stored at 4°C. *Escherichia coli* ATCC25922 (*E. coli*) and *Staphylococcus aureus* ATCC25923 (*S. aureus*) were obtained from China Center of Industrial Culture Collection.

### 2.2 Preparation of GEO films prepared with different surfactants

Ginger essential oil film mixed with different surfactants was prepared on the basis of Liu et al. (2017). Firstly, fish gelatin (8%, w/v) was dissolved in distilled water at 60°C for 90 min. Glycerol was added in gelatin solution (10%, w/w, based on the weight of gelatin), and this solution named mixture A. Then, GEO and surfactant (lecithin/Tween‐20/Tween‐80) were mixed at the ratio of 1:1 (w/w), and this was mixture B. Mixture B was added into mixture A to obtain the GEO concentration of 0.5% (w/v, based on distilled water), and this was mixture C. Mixture C was stirred at 25 ± 2°C for 30 min. Then, mixture C (8 ml) was cast onto a rimmed plastic plate (90 × 90 mm^2^) and dried at 25 ± 2°C and 50 ± 5% relative humidity (RH) for 48 hr. Film without adding surfactant was used as control film. These film samples were prepared for analysis.

### 2.3 The flavor of GEO and the flavor of GEO in films

The flavor of GEO: GEO was diluted to appropriate concentration with dichloromethane solution. The volatile flavor components of GEO were detected by gas chromatography‐mass spectrum (GC‐MS) (Trace1300/ISQ; Thermo Fisher) coupled with the column of HP‐5 (30 m × 0.25 mm × 0.25 μm). The injection volume of GEO diluent was 1 μl, and the initial oven temperature was 40°C, then raised to 290°C at a rate of 6.5°C/min, keeping this temperature for 2 min. The mass detector was carried out in an electron impact mode with ionization energy at 70 eV, and helium gas was selected as carrier gas with the speed of 1 ml/min (Mohamed, El‐Emary, & Ali, 2010).

The flavor of GEO in films: Films were immersed into dichloromethane solution and stirred continuously for 30 min. 1 μl of immersed solution was injected into GC‐MS following the method of GEO test.

### 2.4 Antimicrobial activity

The antimicrobial activity of film was tested on Gram‐negative *E. coli* and Gram‐positive *S. aureus* with the method of Vilela et al. (2017). All the reagents and vessels were sterilized at 121°C for 20 min. The method of plate serial dilution was applied to calculate the bacteria number, and the unit of bacteria number was measured in log cfu/ml. LB medium was used as a bacterial culture medium. The initial bacterial concentration was adjusted to 5 log cfu/ml. 160 mg film was added to LB medium and cultured with bacterial; LB medium without film was used as blank sample. All samples were incubated at 37°C for 24 hr in static condition. The lower value of log cfu/ml demonstrated higher antimicrobial activity. All samples were measured three times.

### 2.5 Physical properties

#### 2.5.1 Film thickness

Digital electronic micrometer (No. 293‐230; Mitutoyo) was used to measure films thickness. Ten random locations were selected as a test point. The values of thickness were used to calculate TS, water vapor permeability (WVP), and opacity.

#### 2.5.2 Water vapor permeability

Water vapor permeability was measured based on ASTM E96‐95 method (ASTM, 1995). The dried CaCl_2_ was placed in glass cups (40 mm wide, 25 mm depth) to keep the RH of inner space 0%. Films were used to seal the glass cup, and then, these cups were placed in desiccator that contains distilled water at 30°C. Weight changes were recorded hourly. WVP was calculated by the following formula:

$$\text{WVP}\;\text{(g}\;m^{-1}\;s^{-1}\;{\text{Pa}}^{-1})=wlA^{-1}t^{-1}{\left(P_{2}-P_{1}\right)}^{-1},$$

where *w* represented the grow weight (g) of the glass cup, *l* denoted the thickness (m) of the film, *A* was the sealed areas (m^2^), *t* was the interval time (s), and (*P*_2_ − *P*_1_) was the difference vapor pressure aside the film (4,244.9 Pa at 30°C). All the films were tested three times to obtain the averaged value.

#### 2.5.3 Mechanical properties

The method of ASTM (2012) was used to examine TS and EAB by Texture Analyzer (Stable Micro System TA.TX‐plus). The trigger force was 5 g, and the test speed was 1 mm/s. Films were cut into strips with 20 mm wide and 50 mm long for experiments. TS and EAB were calculated by the following formula:

$$\text{TS}\;\left(\text{MPa}\right)=F_{\max}/A,$$

$$\text{EAB}\;\left(\%\right)=\left(\Delta L/L_{0}\right)\times 100\%,$$

where *F*_max_ was the maximum force (N) that need to apart the strips, *A* was the cross‐sectional area of the strips (m^2^), Δ*L* was the changed length of strips, and *L*_0_ was the initial length of strips (*L*_0_ = 30 mm). Each type of films was tested for 10 times.

#### 2.5.4 Water solubility

Films were dried at 105°C to obtain a constant weight M_1_, films were immersed into distilled water at 20°C and kept agitating for 24 hr, and then, remained film was dried again at 105°C to obtain a constant weight M_2_; the following formula was used to calculate WS:

$$\text{WS}\;\left(\%\right)=\left(M_{1}-M_{2}\right)÷M_{1}\;\times \;100\%,$$

where *M*_1_ was the initial weight before immersion and *M*_2_ was the weight of retain film after immersion. Each film was tested three times (Rubilar et al., 2013).

#### 2.5.5 Color and opacity

Film's color was tested by colorimeter (CR‐10; Konica Minolta optics Inc). The data were obtained by covering film on the standard white plate. Film's color was represented by the value of *L* (lightness/darkness), a (redness/greenness), b (yellowness/blueness), and Δ*E*; Δ*E* was the total color. The value of Δ*E* was calculated by the following formula:

$$\Delta E=\sqrt{{\left(L-L^{*}\right)}^{2}+{\left(a-a^{*}\right)}^{2}+{\left(b-b^{*}\right)}^{2}},$$

where the value of L*, a*, and b* were came from standard white plate (L* = 92.56, a* = −0.49, b* = −0.25). Each type of film samples was tested eight times (Moradi et al., 2012).

UV‐visible spectrophotometer (Nanodrop200; Thermo Fisher) was used to test the opacity of film. The opacity value was calculated by the formula:

$$\text{Opacity}=A_{600}/x,$$

where *A*_600_ was the absorbance at 600 nm and *x* was the thickness of film (mm) (Wu et al., 2015).

### 2.6 Attenuated total reflectance‐Fourier transform infrared (ATR‐FTIR) analysis

The measurement was conducted by the method of Lin et al. (2019). Films were scanned with an ATR diamond crystal probe at 25°C (Nicolet 6700; Thermo Fisher). The spectra were ranged from 4,000 cm^−1^ to 680 cm^−1^.

### 2.7 Scanning electron microscope

The microstructure of film was analyzed by SEM (S‐3400N; Hitachi). Surface and cross‐section microstructure were obtained according to the method of Nilsuwan, Benjakul, and Prodpran (2018).

### 2.8 Statistical analysis

Statistical tests were performed by Statistical Package for Social Science (SPSS). Duncan test and analysis of variance (ANOVA) were used for analysis, and the difference was considered to be statistically significant if *p* < .05.

## 3 RESULTS AND DISCUSSION

### 3.1 The flavor of GEO and the flavor of GEO in film

The flavor compounds of GEO are illustrated in Table 1. The flavor compounds of GEO were detected to determine whether the flavor of film was affected by GEO flavor. A total of 18 flavor compounds were detected through GC‐MS, including 15 terpenes, 2 aldehydes, and 1 phenol, and these compounds consisted of the flavor of GEO. The mixed flavor of GEO included spicy, woody, herbal, minty, citrus, and another plant flavor. Seven compounds (camphene, α‐myrcene, α‐pinene, α‐phellandrene, copaene, bisabolene, gingerol) contributed to spicy flavor, which accounted for 88.37% of all flavor compounds, whereas only 11.63% of all flavor compounds came from other 11 compounds. It could be speculated that spicy was the main flavor of GEO. The compounds of gingerol had the flavor of spicy, which accounted for 18.42% of all GEO volatile compounds, and accounted for 43.68% of all flavor compounds. Therefore, gingerol might be a characteristic flavor compound in GEO (Karunakaran & Sadanandan, 2019).

**TABLE 1:** The flavor compounds of ginger essential oil

Sequence number	Compound name	Classify	CAS	Molecular formula	Area%	Flavor^a^
1	Hexanal	Aldehyde	66‐25‐1	C_6_H_12_O	0.38	Grass, tallow, fat
2	Camphene	Terpene	79‐92‐5	C_10_H_16_	2.99	Spicy, minty, herbal
3	α‐Myrcene	Terpene	1686‐30‐2	C_10_H_16_	0.33	Spicy, peppery
4	α‐Pinene	Terpene	80‐56‐8	C_10_H_16_	3.77	Spicy, camphor, herbal
5	α‐Phellandrene	Terpene	2243‐33‐6	C_10_H_16_	2.71	Spicy, medicinal
6	1,8‐Cineo	Terpene	470‐82‐6	C_10_H_18_O	1.98	Minty, camphoraceous
8	Linalool	Terpene	78‐70‐6	C_10_H_18_O	0.29	Citrus, floral
9	Borneol	Terpene	507‐70‐0	C_10_H_18_O	0.80	Pine woody camphor balsamic
10	α‐Terpineol	Terpene	98‐55‐5	C_10_H_18_O	0.42	Pine, terpene, lilac, citrus
11	Decanal	Aldehyde	112‐31‐2	C_10_H_20_O	0.47	Sweet, citrus, green melon
12	Copaene	Terpene	3856‐25‐5	C_15_H_24_	0.36	Spicy, wood, honey
14	Bisabolene	Terpene	495‐62‐5	C_15_H_24_	8.68	Spicy, myrrh, citrus, floral,
16	β‐Elemene	Terpene	33880‐83‐0	C_15_H_24_	0.33	Sweet
17	Nerolidol	Terpene	7212‐44‐4	C_15_H_26_O	0.24	Floral, citrus
18	Gingerol	Phenol	23513‐14‐6	C_17_H_26_O_4_	18.42	Spicy
Total					42.17	

^a^ Obtained from http://www.perflavory.com/index.html, http://www.flavornet.org/flavornet.html.

Ginger essential oil flavor compounds in the film are shown in Table 2. It could be noted that two compounds (gingerol and hexanal) in the film were the same as GEO. As a characteristic compound of GEO, gingerol was detected in control film (2.64%) and film prepared with lecithin (0.46%), but it was not detected in the film prepared with Tween‐20 and film prepared with Tween‐80. The detection of gingerol in the film affected the sensory quality of the film. Hexanal had the flavor of grass, tallow, and fat, and the area% of hexanal in each film was close (1.48%–1.57%), while the area% of hexanal in GEO was 0.38%. Hexanal was speculated to be derived from fish gelatin rather than GEO for two reasons: (a) The area% of hexanal in GEO was lower than that of other compounds, and it was difficult to be detected preferentially compared with other compounds with high area%; (b) the resource of gelatin was fish gelatin, and hexanal was a characteristic fish flavor according to previous reports. Liu, Tao, Mccrummen, Hanson, and Wang (2016) found that hexanal was an off‐flavor in catfish fillet; hexanal compounds were observed in fresh fish by Morsy et al. (2016) and Song, Dai, Shen, Peng, and Zhang (2018) considered that hexanal was one of the key volatile compounds of fish oil.

**TABLE 2:** The flavor compounds of ginger essential oil in films

Compound name	CAS	Molecular formula	Area%				Flavor^a^
			Control	Lecithin	Tween‐20	Tween‐80	
Hexanal	66‐25‐1	C_6_H_12_O	1.54	1.48	1.57	1.52	fatty, grassy
Gingerol	23513‐14‐6	C_17_H_26_O_4_	2.64	0.45	n.d.	n.d.	Spicy

Abbreviation: n.d.: not detected.

^a^ Obtained from http://www.perflavory.com/index.html, http://www.flavornet.org/flavornet.html.

Ginger essential oil tended to be accumulated on the surface of control film without the effect of surfactants, and thus, GEO was easily eluted by solvent and detected by GC‐MS. HLB value was the balance between the two‐phase. The HLB value of lecithin, Tween‐20, and Tween‐80 was 4.0, 16.7, and 15.0, respectively; higher HLB value indicated better hydrophilic or polar properties; and lower HLB possessed the opposite characteristics (Schmidts, Dobler, Nissing, & Runkel, 2009). Lecithin was more hydrophobic than Tween‐20 and Tween‐80, and the mixture of GEO with lecithin was difficult to disperse in gelatin solution, and thus, the flavor compounds in GEO were detected in the film; the mixture of GEO with Tween‐20 or Tween‐80, which were hydrophilic, could be dispersed in gelatin solution uniformly; thereby, no GEO compounds were detected in the film. In conclusion, surfactants could reduce the flavor of GEO by dispersing the oil droplet inside the film, and hydrophilic surfactant was more effective on reducing the flavor of GEO compared with the hydrophobic surfactant.

### 3.2 Antimicrobial activity

**FIGURE 1:**
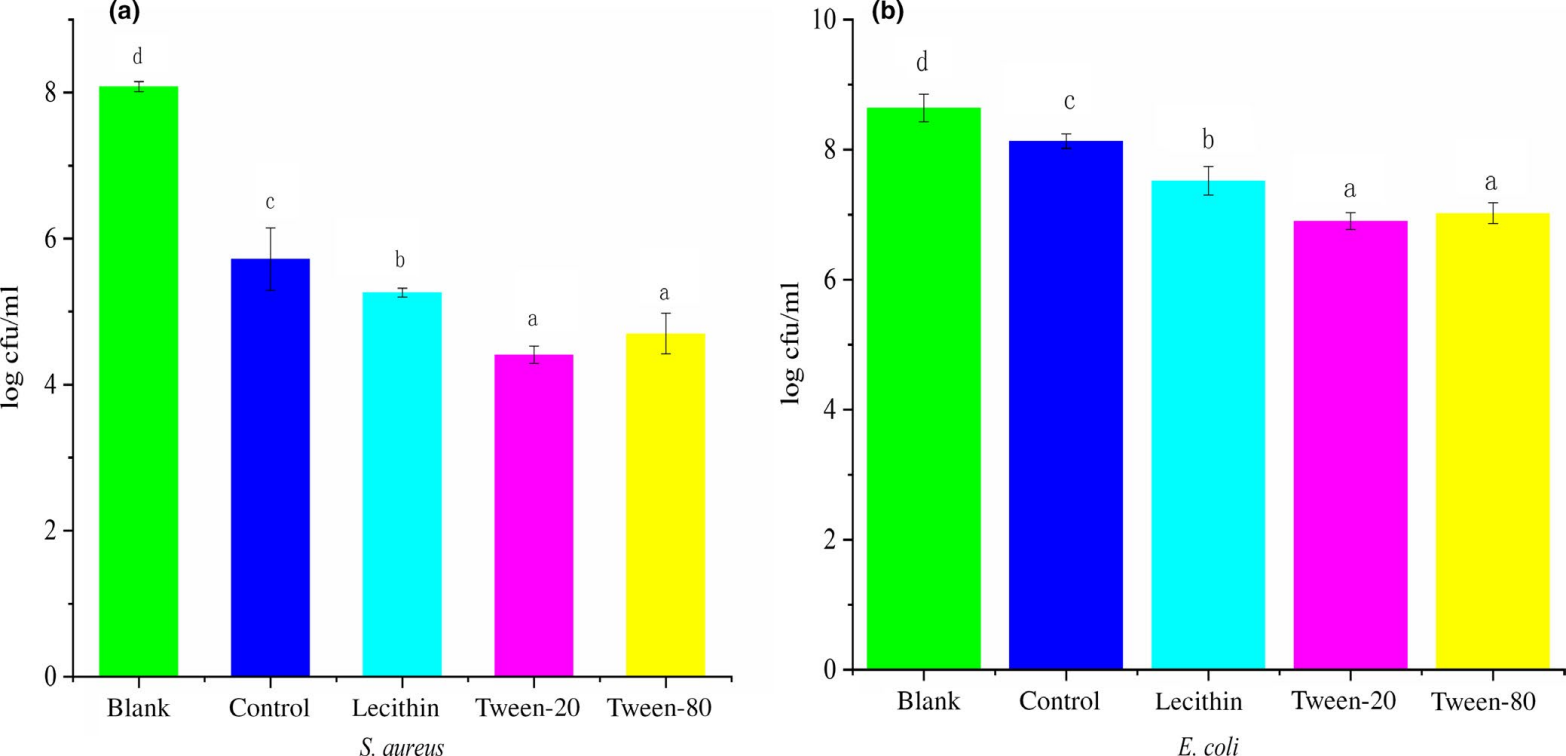
Antibacterial activity of film prepared with different surfactants

The antimicrobial activity of film prepared with different surfactants is exhibited in Figure 1. A lower log cfu/ml value represented a higher antimicrobial ability. All films exhibited antimicrobial activity with various degrees compared with blank group. Control film exhibited the lowest antimicrobial activity of 5.72 log cfu/ml against *S. aureus* and 8.13 log cfu/ml against *E. coli*. After the addition of surfactants into film, the antimicrobial properties of the film increased significantly. Film prepared with lecithin showed antimicrobial activity of 5.26 log cfu/ml against *S. aureus* and 7.52 log cfu/ml against *E. coli*; film prepared with Tween‐20 revealed antimicrobial activity of 4.41 log cfu/ml against *S. aureus* and 6.90 log cfu/ml against *E. coli*; film prepared with Tween‐80 exhibited antimicrobial activity of 4.70 log cfu/ml against *S. aureus* and 7.02 log cfu/ml against *E. coli*. Antimicrobial activity of film prepared with different surfactants was from strong to weak in order: Tween‐20 film, Tween‐80 film, lecithin film, control film.

Ginger essential oil was the main antimicrobial compounds in GEO film, and film prepared with different surfactants showed different antimicrobial activity due to the different dispersion effect of surfactants. The mixture of GEO with hydrophilic surfactant was easily dispersed in gelatin solution. Lecithin was hydrophobic surfactant; the mixture of GEO with lecithin was difficult to disperse in gelatin solution; GEO was easily oxidized, light‐degradation, and evaporation in the process of film‐forming (Buendía‐Moreno et al., 2019); and thus, the antimicrobial activity of film prepared with lecithin increased less. Control film exhibited the lowest antibacterial activity, because GEO would be lost easily without the protection of surfactants.

All films exhibited stronger antimicrobial activity against Gram‐positive *S. aureus* (4.41–5.72 log cfu) than that against Gram‐positive *E. coli* (6.90–8.13 log cfu). A similar result was obtained from Ahmad, Benjakul, Prodpran, and Agustini (2012). GEO was the main antimicrobial compound in the film, and bioactive components in GEO caused cell death through the destructing cell membrane and denaturing protein. Gram‐negative possessed additional external membrane surrounded the cell wall, and it restricted the diffusion of hydrophobic compounds (essential oil) on the membrane; therefore, essential oil had lower antimicrobial activity against Gram‐negative (Oussalah, Caillet, Saucier, & Lacroix, 2007).

### 3.3 Physical properties

#### 3.3.1 Thickness

The thickness of films prepared with different surfactants is shown in Table 3. Control film exhibited the lowest thickness of 0.0704 mm; film prepared with Tween‐20 (0.0760 mm) and film prepared with Tween‐80 (0.0755 mm) had lower thickness than film prepared with lecithin (0.0821 mm); and films prepared with Tween‐20 and Tween‐80 had an approximate thickness of film. Arfat, Benjakul, Prodpran, Sumpavapol, and Songtipya (2014) found that film incorporated with essential oil could prevent film from forming compact and order structure; Ahmad et al. (2012) pointed out compounds in essential oil could interact with gelatin, leading to the protruded structure of the composite film. Surfactants with better dispersion effect contributed to uniform particles of essential oil and eventually due to lower thickness of film. Tongnuanchan, Benjakul, Prodpran, Pisuchpen, and Osako (2016) pointed out the bulky structure of lecithin resulted in more prominent network structure of film compared with film incorporated with Tween‐20/Tween‐80. Therefore, surfactants could increase the thickness of film, and larger structure of surfactants resulted in increased film thickness.

**TABLE 3:** Thickness, water vapor permeability, mechanical properties, and water solubility (WS) of films prepared with different surfactants

Surfactants	Thickness (mm)	Water vapor property (×10^−11 ^gm^−1^s^−1^Pa^−1^)	Tensile strength (MPa)	Elongation at break (%)	WS (%)
Control	0.0704 ± 0.00053 a	7.38 ± 0.52 d	45.33 ± 2.78 c	21.37 ± 2.50 a	14.86 ± 0.33 a
Lecithin	0.0800 ± 0.00139 c	6.03 ± 0.02 a	32.53 ± 2.53 a	44.58 ± 2.61 c	19.29 ± 0.74 c
Tween‐20	0.0760 ± 0.00078 b	7.03 ± 0.30 c	38.56 ± 3.18 b	38.02 ± 2.07 b	16.69 ± 0.94 b
Tween‐80	0.0755 ± 0.00075 b	6.71 ± 0.36 b	38.23 ± 2.14 b	39.13 ± 2.95 b	17.15 ± 0.37 b

Different letters in the same column indicate significant differences (*p* < .05).

#### 3.3.2 Water vapor permeability

Water vapor permeability of films is shown in Table 3, control film exhibited the highest value of WVP (7.38 × 10^−11^ g m^−1^ s^−1^ Pa^−1^), and the WVP of film prepared with lecithin (6.03 × 10^−11^ g m^−1^ s^−1 ^Pa^−1^) was lower than the films prepared with Tween‐20 (7.03 × 10^−11^ g m^−1^ s^−1^ Pa^−1^) and Tween‐80 (6.71 × 10^−11^ g m^−1^ s^−1^ Pa^−1^). Surfactants could contribute to the uniform distribution of the lipid in the film matrix, which were beneficial to promote the moisture resistance of film (Dickinson, 2003; Xiao et al., 2016). Thus, film incorporated with surfactants possessed lower WVP than control film, regardless of the type of surfactants. Higher HLB value represented better hydrophilic or polar properties, and lower HLB possesses the opposite characteristics (Schmidts et al., 2009). Adding nonpolar and hydrophobic materials would reduce the absorptivity and diffusivity of water vapor, due to decreased WVP (Arfat et al., 2014; Xiao et al., 2016). Thus, film prepared with lecithin had lower WVP value than films prepared with Tween‐20 and film prepared with Tween‐80. In conclusion, film prepared with different surfactants had prominent difference in WVP, and the WVP values of film were ranked from high to low: control film, Tween‐20 film, Tween‐80 film, lecithin film.

#### 3.3.3 Mechanical properties

Tensile strength and EAB are shown in Table 3, and these two parameters were used to evaluate the mechanical properties of film. Control film possessed the highest TS (45.33 MPa) and the lowest EAB (21.37%) among all films. The value of TS decreased and the value of EAB increased with the addition of surfactants, the film prepared with lecithin had the lowest TS (32.53 MPa) and the highest EAB (44.58%); and comparatively, film prepared with Tween‐80 had TS = 38.56 MPa and EAB = 38.02% and with Tween‐20 had TS=38.23 MPa and EAB=39.13%. Tongnuanchan, Benjakul, and Prodpran (2012) had the opinion of that interaction between nonpolar molecules (lipid) and interaction between polar polymer and nonpolar molecules became weaker compared with those between polar polymer molecules. Limpisophon, Tanaka, and Osako (2010) proposed oil or lipids in the protein‐based film might affect the interactions between polymer chains by providing the flexible region of the film. Thus, the addition of essential oil in film resulted in the reduced TS and increased EAB of film. The role of surfactant was to promote the dispersion of oil droplets in protein solution and reduce the interaction between protein molecules, eventually leading to the decreased TS and increased EAB.

The difference in hydrophilic/hydrophobic properties of surfactants would influence the mechanical properties of the film. Lecithin was more hydrophobic than Tween‐20 and Tween‐80. Adding hydrophobic compounds to polymer network would form heterogeneous structure and discontinuous areas in film, which would eventually lead to a decreased TS of the film (Bravin, Peressini, & Sensidoni, 2004). Therefore, the TS value of lecithin film was lower than that of Tween‐20 film and Tween‐80 film. In addition, Andreuccetti, Carvalho, Galicia‐García, Martínez‐Bustos, and Grosso (2011) proposed that larger molecules would hinder effective inclusion in the polymer matrix, resulting in a longer elongation of film. Lecithin with bulky structure resulted in higher EAB value of film compared with that of Tween‐20 film and Tween‐80 film (Tongnuanchan et al., 2016). A similar phenomenon was observed in the preparation of gelatin film using extract/lecithin as surfactants, yucca extract was less hydrophobic than lecithin, and film prepared with lecithin possessed lower TS and higher EAB compared with film prepared with yucca extract (Andreuccetti et al., 2011). There was no significant difference in mechanical properties between Tween‐20 film and Tween‐80 film, because the hydrophilicity of Tween‐20 and Tween‐80 was similar.

#### 3.3.4 Water solubility

Water solubility of films incorporated with different surfactants is illustrated in Table 3. Control film possessed the lowest WS (14.86%); WS increased with the addition of surfactants, and film prepared with lecithin had the highest WS (19.29%); the value of WS in Tween‐20 film (16.69%); and Tween‐80 film (17.15%) was close. WS reflected the film's resistance to water, film with low WS helped to maintain the integrity of the food and extended the shelf life of the food. WS was affected by the internal structure of film. Film incorporated with surfactants formed coarseness surface and loose structure, which would increase the contact area between the film and water, enhancing the WS of film (Song, Zuo, et al., 2018). Film prepared with lecithin had looser structure and higher WS due to the bulky structure of lecithin.

#### 3.3.5 Color and opacity

Color of film is expressed through the value of Δ*E*, *L*, *a*, and *b*. As exhibited in Table 4, control film possessed the lowest *b* value (−6.26), while the highest Δ*E* value (6.74), *L* value (91.10) and *a* value (2.18); with the addition of surfactants, *b* value increased, while Δ*E* value, *L* value and *a* value decreased; film prepared with Tween‐20 and film prepared with Tween‐80 had proximate value in Δ*E* *L* and *b*. Tween‐20 and Tween‐80 were light yellow liquid, lecithin was light yellow solid, and *b* value of films was increased upon the addition of these surfactants. Δ*E* value was calculated by *L*, *a* and *b*, film with surfactants treatment had lower Δ*E* value compared with the control film.

**TABLE 4:** Color and opacity of films prepared with different surfactants

Surfactants	Δ*E*	*L*	*a*	*b*	Opacity
Control	6.74 ± 0.75 c	91.10 ± 0.13 c	2.18 ± 0.05 d	−6.26 ± 0.05 a	1.84 ± 0.05 a
Lecithin	4.44 ± 0.16 b	90.44 ± 0.09 a	1.64 ± 0.05 a	−3.51 ± 0.22 b	8.96 ± 0.17 c
Tween‐20	3.83 ± 0.10 a	90.58 ± 0.10 b	1.85 ± 0.08 b	−2.54 ± 0.13 c	7.27 ± 0.14 b
Tween‐80	3.80 ± 0.08 a	90.56 ± 0.09 b	1.91 ± 0.06 c	−2.43 ± 0.10 c	7.18 ± 0.07 b

Different letters in the same column indicate significant differences (*p* < 0.05).

Opacity of films at 600 nm is shown in Table 4, and control film had the lowest opacity (1.84) among all films; opacity of film increased with the addition of surfactants, and film prepared with Tween‐20 (7.27) and film prepared with Tween‐80 (7.18) had lower opacity than film prepared with lecithin (8.96). The transparency and light scattering intensity of the films were related to the oil droplet size of the dispersed phase. As the size of oil droplets increased, the intensity of light scattering was enhanced due to lower transparency and higher opacity of the film (Monedero, Fabra, Talens, & Chiralt, 2009; Nur Hanani & Aelma Husna, 2018; Sánchez‐González, Vargas, González‐Martínez, Chiralt, & Cháfer, 2009). Uniform structure in protein solution was formed easily by hydrophilic surfactant compared with that by hydrophobic surfactant (Song, Zuo, et al., 2018; Sothornvit, Rhim, & Hong, 2009). Lecithin had weak dispersion effect than Tween‐20 and Tween‐80, and film prepared with lecithin likely formed nonuniform oil droplets in film due to the enhanced intensity of light‐scattering by larger size of oil droplets; thus, opacity of film prepared with lecithin became higher. A similar result was obtained from Andreuccetti et al. (2011), and film prepared with lecithin had higher opacity than that of film prepared with yucca extract, which was a hydrophilic surfactant. No significant difference in opacity was achieved between film prepared with Tween‐20 and film prepared with Tween‐80 because of the similar hydrophilic property of Tween‐20 and Tween‐80.

### 3.4 Attenuated total reflectance‐Fourier transform infrared (ATR‐FTIR) analysis

**FIGURE 2:**
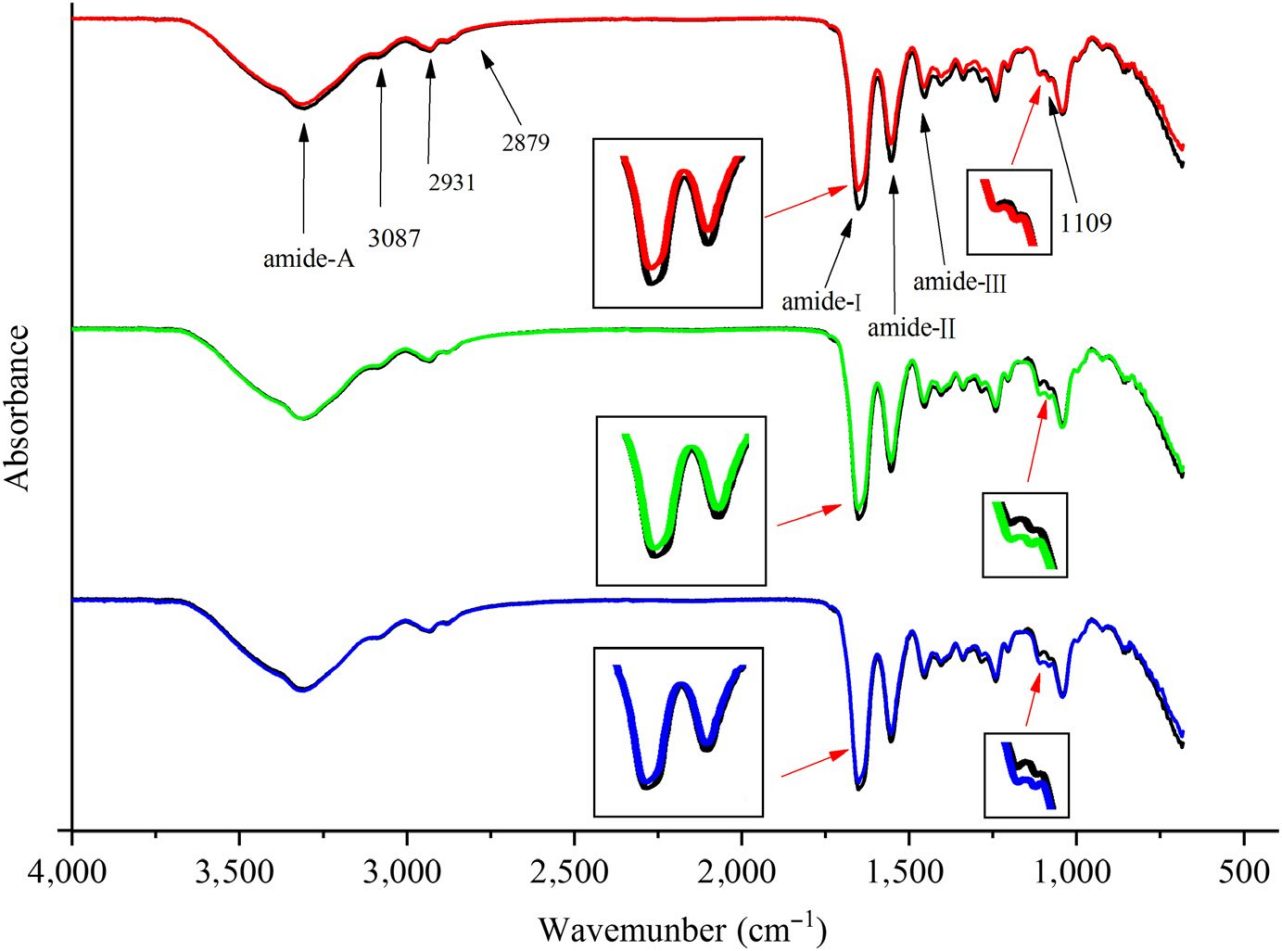
ATR‐FTIR analysis of film prepared with different surfactants

ATR‐FTIR spectra of films prepared with different surfactants are illustrated in Figure 2, and amide A located at 3,305–3,307 cm^−1^ represented N–H stretching coupled with hydrogen bonding (Muyonga, Cole, & Duodu, 2004). The wavelength at 3,087–3,091 cm^−1^ represented C–H stretching coupled with NH stretching. The band at 2,879 cm^−1^ and 2,931 cm^−1^ demonstrated asymmetrical and symmetrical stretching vibrations of the aliphatic C–H in CH_2_ and CH_3_ (Guillén & Cabo, 2004; Muik, Lendl, Molina‐Diaz, Valcarcel, & Ayora‐Cañada, 2007; Tongnuanchan et al., 2012). Amide I, amide II, and amide III were located at 1,651 cm^−1^, 1,552 cm^−1^, and 1,242 cm^−1^, respectively. Amide I illustrated the C=O stretching/hydrogen bonding coupled with COO–, amide II represented bending vibration of the N–H groups and the stretching vibration of the C–N groups, and amide III illustrated the N–H bending (Jackson, Choo, Watson, Halliday, & Mantsch, 1995). The vibration at 1,109–1,111 cm^−1^ illustrated the C–O stretching vibration of the ester group (Guillén & Cabo, 2004), and the existence of C–O suggested a covalent interaction between protein and essential oil. In addition, film prepared with lecithin exhibited lower amplitude at amide I compared with the film prepared with Tween‐20 and film prepared with Tween‐80, which illustrated that film prepared with lecithin had weaker hydrogen bond; film prepared with Tween‐20 and film prepared with Tween‐80 had higher amplitude at 1,109–1,111 cm^−1^, this change suggested that covalent interaction was enhanced.

### 3.5 Scanning electron microscope

**FIGURE 3:**
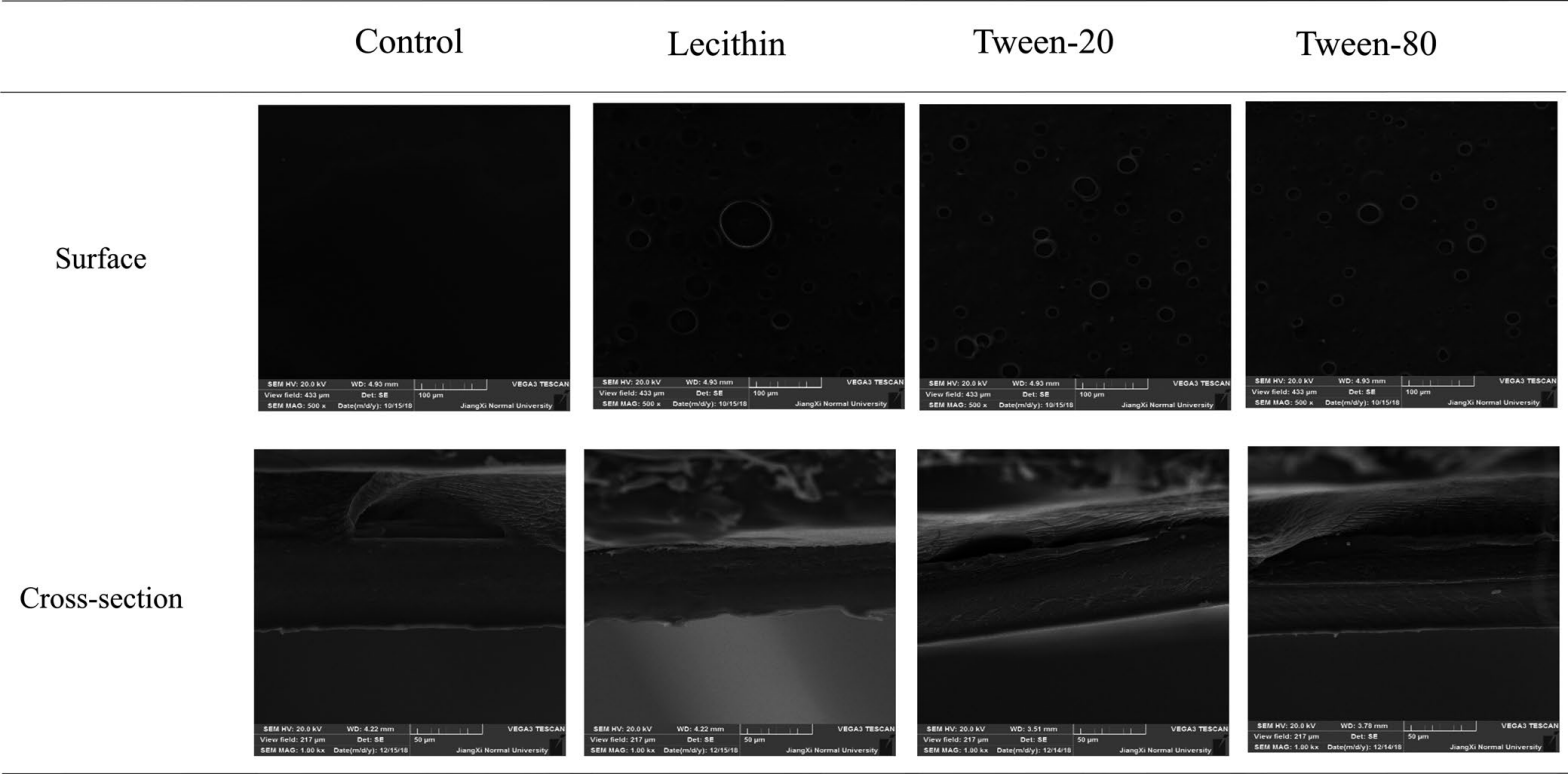
Surface and cross‐sectional micrographs of film prepared with different surfactants

The surface morphologies and freeze‐fractured cross‐section morphologies of film incorporated with different surfactants are shown in Figure 3. In terms of surface morphologies, control film exhibited confirm and smooth surface with faint oil stain. After the addition of surfactants, oil droplets were appeared in film; oil droplets presented in Tween‐20 film and Tween‐80 film were uniform; and oil droplets presented in lecithin film were nonuniform. In cross‐sectional morphologies, control film was compacted and drape, and loose structure of films was formed by the addition of surfactants.

The structure of film was related to the properties of film. The structure of control film was firm and smooth, and thus, control film possessed the lowest thickness, EAB and WS, and the highest thickness, TS. Essential oil in film hindered the interaction between proteins upon the addition of surfactants, composite film formed looss structure and flexible region, due to increased thickness, EAB and WS, decreased TS.

### 3.6 Schematic model

**FIGURE 4:**
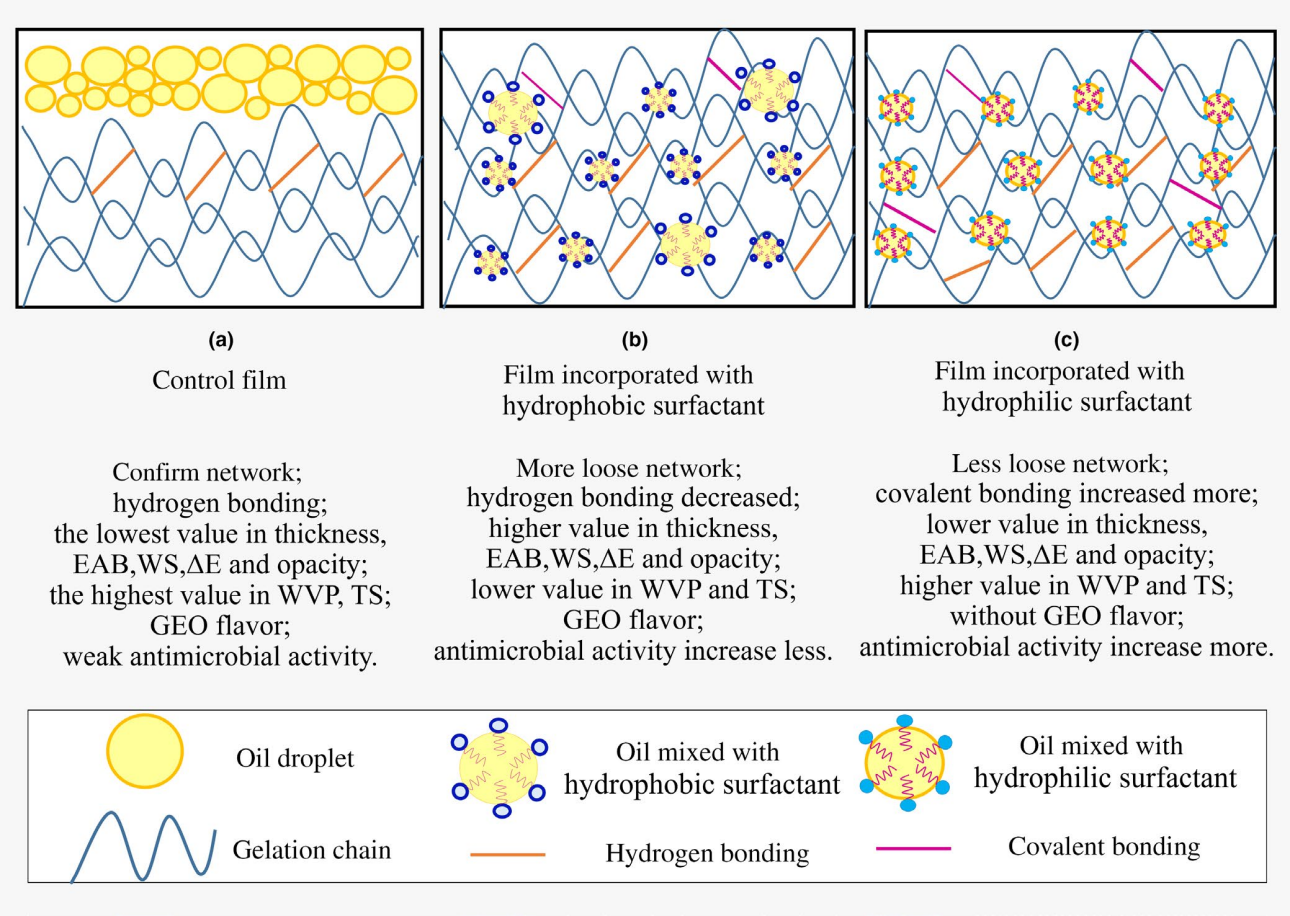
Simplified illustration of the change in the properties of film prepared with different surfactants

In general, control film without adding surfactant (Figure 4a) exhibited the lowest value of thickness, WS, EAB, Δ*E*, and opacity, while the highest value in WVP and TS, and flavor compounds of GEO were detected, and the antimicrobial activity was weak. After the addition of hydrophobic surfactant (Figure 4b), film formed loose network, hydrogen bond strength decreased slightly; the value of thickness, EAB, WS, Δ*E*, and opacity was increased remarkably, while the value of WVP and TS was decreased significantly. Flavor compounds of GEO were detected, and the antimicrobial activity was lower. With the addition of hydrophilic surfactant (Figure 4c), loose network of film was formed, covalent bond strength increased slightly; the value of thickness, EAB, WS, Δ*E*, and opacity increased less, while the value of WVP and TS decreased less, flavor compounds of GEO were not detected, and the antimicrobial activity was higher.

## 4 CONCLUSION

The role of surfactants was to promote the dispersion of oil droplets in gelatin solution, and hydrophobic surfactants had poor dispersion effect in gelatin solution compared with hydrophilic surfactants. Film incorporated with lecithin (hydrophobic) had lower value of WVP and TS, while higher value of thickness, EAB, WS, Δ*E* and opacity, and flavor of GEO could be detected, and the antimicrobial activity increased less. Film prepared with Tween‐20 and film prepared with Tween‐80 (hydrophilic) had higher value of WVP and TS, while lower value of thickness, EAB, WS, Δ*E* and opacity, and the flavor of GEO was not detected, and the antimicrobial activity increased more. Film incorporated with Tween‐20 and film incorporated with Tween‐80 had approximate properties except WVP, because the HLB value of Tween‐20 and Tween‐80 was close.

## CONFLICT OF INTEREST

The authors declared that they have no conflicts of interest in this work.

## ETHICAL APPROVAL

The study did not involve human and animal testing.
